# Time Devours Things: How Impulsivity and Time Affect Temporal Decisions in Pathological Gamblers

**DOI:** 10.1371/journal.pone.0109197

**Published:** 2014-10-08

**Authors:** Alessandro Grecucci, Cinzia Giorgetta, Andrea Rattin, Cesare Guerreschi, Alan G. Sanfey, Nicolao Bonini

**Affiliations:** 1 Department of Psychology and Cognitive Sciences, University of Trento, Trento, Italy; 2 Institute of Cognitive Science and Technology, Consiglio Nazionale delle Ricerche, Rome, Italy; 3 Società Italiana Intervento Patologie Compulsive (SIIPAC), Bolzano, Italy; 4 Department of Psychology, University of Arizona, Tucson, Arizona, United States of America; 5 Donders Institute for Brain, Cognition and Behavior, Radboud University Nijmegen, Nijmegen, The Netherlands; 6 Department of Economics and Management, University of Trento, Trento, Italy; Swansea University, United Kingdom

## Abstract

Impulsivity is associated with several psychiatric disorders in which the loss of control of a specific behavior determines the syndrome itself. One particularly interesting population characterized by reported high impulsivity and problematic decision-making are those diagnosed with pathological gambling. However the association between impulsivity and decision making in pathological gambling has been only partially confirmed until now. We tested 23 normal controls and 23 diagnosed pathological gamblers in an intertemporal choice task, as well as other personality trait measurements. Results showed that gamblers scored higher on impulsivity questionnaires, and selected a higher percentage of impatient choices (higher percentage of smaller, sooner rewards), when compared to normal controls. Moreover, gamblers were faster in terms of reaction times at selecting the smaller, sooner options and discounted rewards more rapidly over time. Importantly, regression analyses clarified that self-reported measures of impulsivity played a significant role in biasing decisions towards small but more rapidly available rewards. In the present study we found evidence for impulsivity in personality traits and decisions in pathological gamblers relative to controls. We conclude by speculating on the need to incorporate impulsivity and decision biases in the conceptualization of pathological gambling for a better understanding and treatment of this pathology.

## Introduction

Impulsivity is a prominent feature of several psychiatric disorders in which the loss of control of a specific behavior determines the syndrome itself, such as: substance dependence [1], attention-deficit/hyperactivity disorder (ADHD) [2], and pathological gambling [3]. Furthermore, higher impulsivity has also been linked to higher rates of relapse in substance users, individuals with bipolar disorder, and more importantly with the present paper, pathological gamblers [4], [5]. Notably, these pathological deviations may reflect an exaggeration of basic personality traits that are present in the non-pathological population [6], [7].

Although the term “impulsivity” is widely used, there is no general agreement in terms of its broader definition. Some authors suggested that the concept of impulsivity may be considered as an umbrella term with different facets that may be linked to a clinical condition (see also the concept of positive and negative urgency in [8]. Beside general personality trait definitions, factor analyses of neuropsychological measures (Go/No Go, Stroop task, Stop Signal task) have revealed the existence of an “inhibitory control” system that when damaged leads to impulsive behavior [3]. The lack of inhibition can be also seen as a lack of self-control [9], or diminished self-regulation [4]. Importantly, individual differences in lack of inhibition or self-control can explain individual differences in impulsive behaviors for both normal and pathological populations [10].

One particularly interesting population characterized by reported high impulsivity and problematic decision behavior is the one diagnosed with pathological gambling. Pathological gambling afflicts about 2% of the general population [11] and was classified until now (DSM IV-TR, APA, 1994) as a disorder of impulse control, though in the actual version (DSM V, APA, 2013) it has been classified under the substance related and addictive disorders. However, the vast majority of studies have found that gamblers score higher than control participants on personality inventories assessing impulsivity [12], [13], [14], [15], [16]. Previous studies have found diminished neurocognitive self-regulatory functions in PG [17], and neurological changes in reward systems [18], [19]. Neurobiological studies indicate that diminished dopamine receptor availability (due to addiction behaviors) may cause a chronic reward deficiency in the brain, resulting in a vulnerability towards reward dependent behaviors [20], [21].

One way to test for impulsivity in abnormal populations is to use self-reported measures such as questionnaires that measure thoughts and behaviors related to impatience and lack of control. This kind of measurement can be viewed as an indicator of the phenotype of the disorder [4]. Another way is represented by performance measure of reward sensitivity/processing that detect underlying problems in basic cognitive functions such as decision making. This kind of measurement can be seen as an indicator of the endophenotype of the disorder, or the functions that underlie a disorder [4], [22]. Of all kinds of decision that we are daily asked to make, there is one of particular interest for the present paper known as intertemporal decisions, strictly related to impulsivity and impatience. Intertemporal choices refer to choices that are available at different time points in which one has to evaluate the trade-off between costs and benefits of waiting to have that option. When making intertemporal decisions, humans tend to prefer the soonest available option even if it is the smallest one. Behavioral economists have proposed a discounted utility model (DUT) that relies upon an normative framework to account for intertemporal choices [23]. For example, according to Mazur [24] the value of a reward decreases over time with a hyperbolic function: SV = R/(1+ kT), where SV is the subjective value of the delayed reward R after a waiting time T, and k is the delay discount rate. Though this model has been constructed to capture more precisely people’s decisions, it assumes that decision-makers choose between options based on a weighted sum of utilities with the temporal discount factor as a weight. Therefore according to this model, humans should show a preference for the option that maximizes utility across a reasonable waiting time. However, this is not always the case. Indeed, humans can be very impatient when making a monetary choice, thus violating the DUT (i.e. exhibiting a steeper hyperbolic function than Mazur equation predicts). These time-inconsistent preferences have been shown in the animal and human literature for several decades. A common Latin saying is “tempus edax rerum” (Ovidio, Metamorfosi, XV, 234) that is, “time devours things”. This intuition takes account of our tendency to incorporate in valuation both the stimulus itself (the reward) as well as how long it will take for us to receive it. Reward is an important and ubiquitous aspect of decision-making. Intertemporal decisions seem highly related to impulsivity [25], and are often used as a measure of trait impulsivity [26]. Indeed, temporal discounting is thought to rely on two separate processes, a logical/rational and an emotional/visceral process [27]. The degree to which one chooses the emotionally relevant response (i.e., choosing an immediate reward) may be determined by the lack of control over immediately available rewards, or in other words by impulsivity. Animal studies across species [28], [29], [30], [31], [32], [33], [34] have also shown a preference for the small immediate reward, at least once the large reward delay exceeds some threshold limit. Beside animals, also humans typically tend to prefer choosing the immediately available reward. Indeed, when choosing between an immediate and a delayed reward, both humans and animals typically tend to prefer choosing the immediately available reward, even if it is sometimes substantially smaller than the delayed option [35], [36].

Several factors can potentially affect this time related devaluation. Some authors pointed out that the degree to which one chooses the immediate reward may be determined by diminished neurocognitive self-regulatory functions, or in other words by impulsivity [37], [4], [38], [27]. Diminished self-regulation means that an addicted person is not able to inhibit the urge for a desired drug or behavior [4]. If this is the case, we argue that people with severe lack of impulse control (i.e. high impulsive traits) are much more impatient in their choices, i.e. prefer the soonest available reward even though this is the less monetarily valuable.

In the recent past there have been a few attempts to study impulsive decision-making in populations suffering from addiction. Impatient choices have been evaluated in substance abusers (another syndrome characterized by lack of control over immediate rewards, i.e. heroin), and authors found that these populations discounted delayed monetary rewards more rapidly than controls [39]. Similar results were found for cigarette smokers [40], and for heavy drinkers [41].

More relevant to the present paper, a study from Holt, Green, and Myerson [42] reported significantly steeper discount function in student gamblers when compared with a matched control group. However, this study did not involve real pathological gamblers. A first attempt to study impulsive decisions in pathological gamblers was conducted by Petry [43], who tested subjects with gambling problems in intertemporal scenarios. Using gamblers both with and without substance use disorder, they found that gamblers with substance use disorder discounted more rapidly rewards over time when compared with both controls and pure gamblers (respectively: k = 0.30, k = 0.02, k = 0.07). However, it is well known that substance use has neurotoxic effects on the brain and indeed may have been the primary causes of the results, rather than gambling behavior itself. Moreover, the pure gamblers used in this study were not an actual clinical population, but were rather recruited through advertisements for free and confidential gambling treatment. The experiment consisted of a scenario of printed cards with two options randomly selected per subject. This scenario methodology has been criticized as in some cases people may be less susceptible to emotional involvement than when they perform an actual choice [44]). Further, results on actual choices, reaction times, and eventual relationships between actual choices and impulsivity were not presented, though a correlation with all k values and Eysenck test was shown. Another study by Brevers and colleagues [45], studied delay discounting in pathological gamblers finding stronger discount rates in gamblers relative to controls. However, the gamblers tested in this study showed other differences in relevant pathological dimensions, rather than gambling addiction only, such as anxiety (but also depression and ADHD), though limiting the interpretation of the results. Previous studies showed that anxiety may strong bias decision making [46], [47]. Moreover, even in this study, actual choices, reaction times, and eventual relationships between actual choices and impulsivity were not considered.

In another study, Alessi and Petry [48], replicated previous results on steeper discounting, but this time evaluating the role of impulsivity in intertemporal choices, and found that gambling severity was the best predictor (soon followed by impulsivity measures) of impulsive choices. Finally, a paper from Dixon and colleagues [49], reported steeper discount rates for gamblers when tested in gambling versus non gambling contexts. However, these studies did not involve a control group, and the group of gamblers reported comorbidity for other addictions (alcohol and drug).

The aim of this paper is to examine the inherent impulsivity of the decision-maker as a potential factor that leads to making impatient choices in pathological gamblers as compared to normal controls. Scholars usually conceptually separate the phenotype of a disorder (the phenomenological level) from the endophenotype (the functions that underlie the disorder) [4]. To study the first level, self-report measures are used, whereas indicators of the second level are behavioral (e.g. reaction times) and physiological indexes (e.g. electroencephalography), indexes that are able to detect hidden features behind the appearance of the disorder. Though correlated, scholars showed different predictive values of these two kinds of measures [50]. In the present study we used self-administered impulsivity questionnaires (BIS11 and PG-Y-BOCS) as phenotypical measures of impulsivity, and intertemporal decision task, as an endophenotypical measure [4]. We predict differences at both levels in the two populations. We predict gamblers will show a higher percentage of smaller sooner choices, faster responses (in the neurocognitive task), and higher impulsivity traits. In other words, we aim to show a clear relationship between impulsivity and choices (in the self-report measures). We also expect that time (in the form of longer delays) bias pathological gamblers decisions towards the more impatient choice and that they overall discount values more faster than controls. Finally, we expect a relationship between phenotypical and endophenotypical measures of impulsivity.

## Methods

The ethical review board of the University of Trento approved the study and written informed consent was obtained from all participants.

### 2.1 Participants

Twenty-three adults (20 males, 3 females; mean age = 37.47, *SD* = 9.36; education = 12.65, *SD* = 1.92) diagnosed with pathologic gambling in line with DSM IV (American Psychiatric Association, 1994) and ICD 10 (World Health Organization, 1994) criteria, and twenty-three controls (20 males, 3 females; mean age = 35.61, *SD* = 9.00; education = 12.09, *SD* = 2.96) without physical, psychiatric or neurological problems were recruited in the present study. Patients were recruited from a clinical centre devoted to the treatment of addictions known as S.I.I.Pa.C (Società Italiana di Intervento Patologie Compulsive) in Bolzano, Italy. Importantly, patients with substance abuse and other comorbid disorders (such as anxiety disorders), or cognitive impairment, were excluded from the study.

### 2.2 Experimental Procedures

#### a) Assessment

Gambling severity was assessed for both populations by the South Okas Gambling Screen questionnaire (SOGS [51]). In addition, participants also filled out a series of self-administered questionnaires in the assessment phase (before the experimental phase in order to avoid any carryover effect). These comprised the Positive and Negative Affective scales (PANAS, trait version; [52]) to test for the level of negative affect; the State-Trait Anxiety Inventory (STAI 1 and 2, [53]), to test for anxiety traits. Notably, these gamblers suffered mainly from common non-strategic gambling (slots, video poker, cards).

#### b) Self report measures of impulsivity

The Barratt Impulsiveness Scale (BIS; [54]) was administered to test for participants’ trait level of general impulsivity, as well as the Pathological Gambling Yale Brown Obsessive Compulsive Scale (PG Y-BOCS; [55]), to assess participants’ impulsivity and compulsivity in gambling. These two scales can differentiate the participants on impulsivity traits, and can be used to test our hypothesis regarding a link between impulsivity and impatient choices.

#### c) Performance measure of reward sensitivity/processing

**Figure 1:**
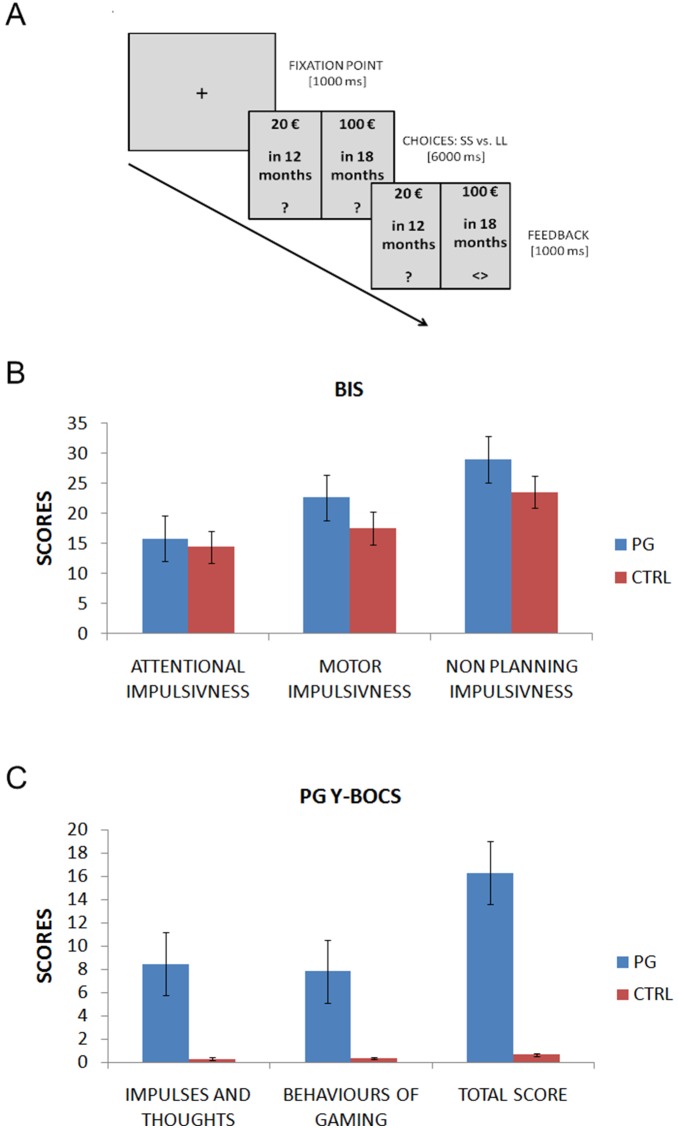
Experimental procedure and self-report measures. (A) A timeline of the experiment is presented. See text for further information. (B) Results from the Barratt Impulsivity Scale (BIS), measuring impulsivity traits are displayed. Gamblers scored higher level of impulsivity as compared with controls, over two subscales (Motor impulsiveness and non- planning impulsiveness). (C) PG-Y-BOCS scale also showed larger differences across the two populations, with gamblers scoring much higher on both subscales and on the total score scale.

Participants were first instructed as to the nature of the task (see Figure 1A for details). They were told the study aimed to assess how people make decisions about different monetary rewards, which can be obtained after a shorter or longer waiting time. They were told that every trial consisted of one option with a relatively small monetary reward obtainable after a short delay, and a larger monetary reward obtainable after a longer delay.

The computerized task consisted of 194 trials, divided into 4 sessions of 48 choices. Within each block the following variables were manipulated: Waiting time for shorter sooner (SS) options: today, 1 month, 6 months, 12 months and 18 months; Waiting time for longer later (LL) options: today, 1 month, 6 months, 12 months, 18 months and 24 months; Reward values for SS options: 5 €, 10 €, 20 €, 30 €, 40 €, 50 €, 60 €, 70 €, 80 €, 90 €, 95 €, 100 €; Reward value for LL options: 100 €. Inside each block, every amount from 5 € to 95 € was repeated four times (two times per left and right side). The 100 € offer was repeated 26 times per side. Moreover, every time delay was repeated 8 times per side. In total, there were 96 offers paired in 48 choices for every block. Importantly both groups were administered with the same type and number of offers. The four blocks were counterbalanced and had the same amount of time and money. Participants were instructed as to how to select the preferred option by pressing one of two buttons within a time limit of 10 seconds. After the choice, a feedback signal for the selected option appeared. Importantly, participants were informed they would be paid with real money, based on a choice randomly selected at the conclusion of the experiment. Pairs of stimuli were displayed randomly, with each combination repeated 4 times and counterbalanced across the left-right side of the screen (see Figure 1A).

Before running the experiment subjects performed a training session consisting of 8 trials with options similar to those shown in the experimental phase.

## Results

### 3.1 Assessment

First of all, no significant differences between the groups in terms of age (*p* = .51) and education (*p* = .58) were visible (see Table 1). As expected, patients and controls differed significantly in terms of gambling severity (SOGS, PG scored: 12.30, CTRL scored: 0.13, *t*(44) = 17.55, *p*<.001); but not in anxiety STAI1 (*t*(44) = 1.58; *p* = .121) and STAI2 (*t*(44) = 0.76; *p* = .449), excluding the possibility of comorbid pathologies (Giddens et al., 2012). The Positive and Negative Affective scales (PANAS, trait version) demonstrated a significant difference in the “Negative Affect” scale (gamblers: 20.70, controls: 14.91 *t*(44) = 3.72, *p*<.001), but not in “Positive Affect” (*p*>.05).

**Table 1:** Demographic and clinical assessment.

	Pathological gamblers	Normal controls	Sig.
**a) Demographic data**			
**Age**	35.61 (9.00)	37.48 (9.94)	p = 0.51
**Education**	12.13 (2.69)	12.65 (1.92)	p = 0.48
**Sex**	20 M, 3 M	20 M, 3 F	/
**Ethnicity**	All caucasian	All caucasian	/
**Handedness**	21 right, 2 left	20 right, 3 left	/
**b) Diagnostic data**			
**Diagnosis (DSM IV)**	23 PG	Without history of neurological and psychiatric diagnosis	/
**SOGS**	12.30 (3.27)	0.13 (0.63)	p<.0001
**PANAS - positive affect**	29.39 (6.48)	31.52 (7.30)	p = .30
**PANAS - negative affect**	20.70 (6.70)	14.91 (3.29)	p<.0001
**STAI 1**	38.48 (5.70)	41.22 (5.11)	p = .12
**STAI 2**	35.48 (7.83)	37.17 (7.22)	p = .45

### 3.2 Self report measures of impulsivity

Importantly for the present study, the two groups differed in the BIS “motor impulsiveness” subscale (PG: 22.70, CTRL: 17.57, *t*(44) = 4.17, *p*<.0001), measuring a relative lack of control in motor behavior, and in the BIS “non-planning impulsiveness” subscale (PG: 29.04, CTRL: 23.56, *t*(44) = 5.29, *p*<.0001), indicating a deficit in planning their behavior, but not in the BIS “attentional impulsiveness scale” (*t*(44) = −1.623; *p* = .112). Moreover, the groups differed in terms of impulsivity and compulsivity at gambling in the PG-Y-BOCS (obsession subscale: PG: 8.48, CTRL: 0.30, *t*(44) = 9.01, *p*<.0001, and in compulsiveness subscale PG: 7.83, CTRL: 0.35, *t*(44) = 7.61, *p*<.0001). See Table 1 and Figure 1. These results confirm that PG have higher impulsivity in self-reported measures (phenotypical level).

### 3.3 Performance measure of reward sensitivity/processing

#### a) Choices

**Figure 2:**
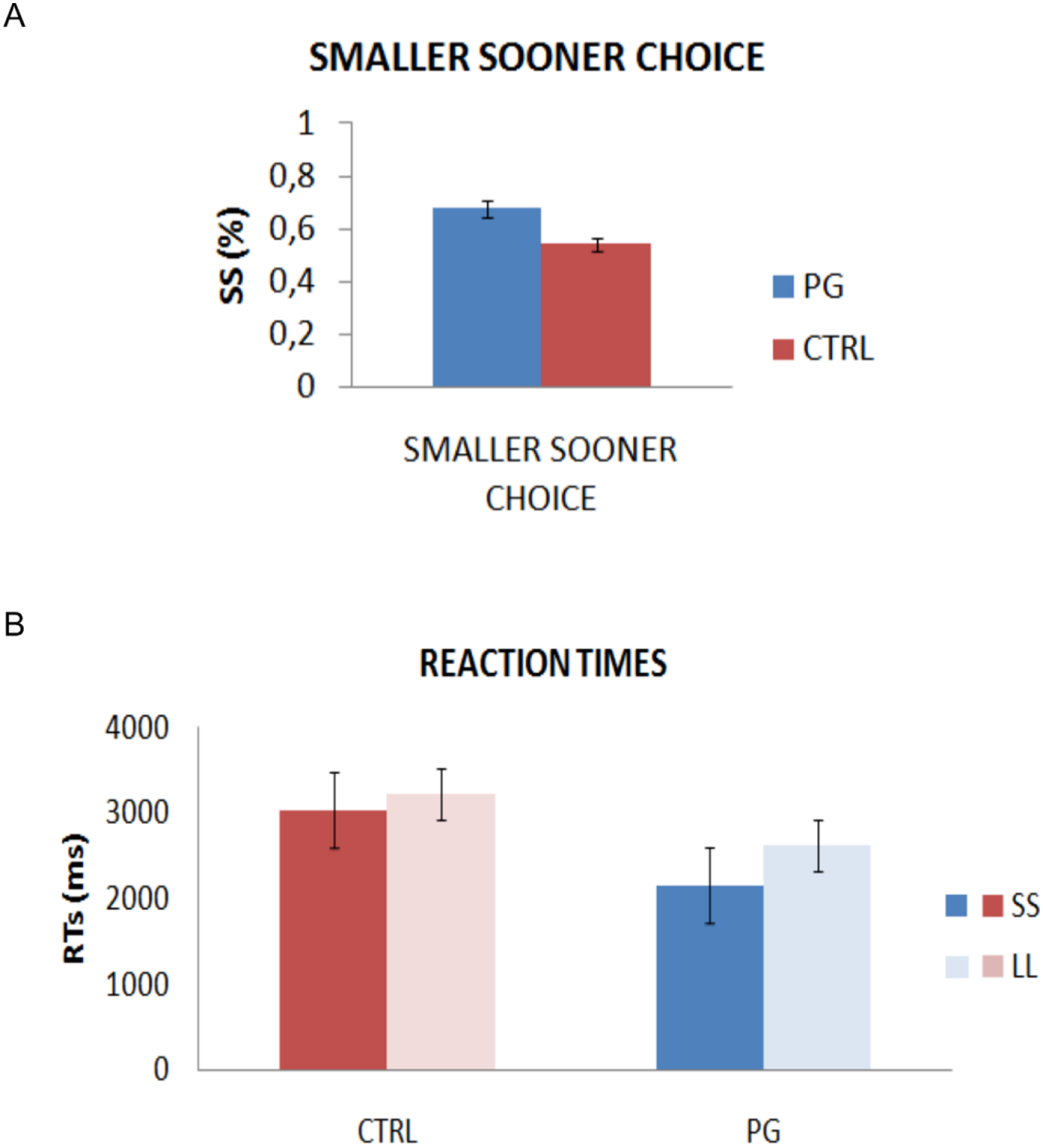
Performance results. (A) The percentage of smaller sooner choices statistically differed across the two populations, with gamblers showing higher percentage of SS. (B) Reaction times analyses showed interesting differences between the two populations with gamblers being faster in selecting SS choices only. Bars indicate stanmdard error of the mean.

To examine differences in choices between patients and controls, we compared the number of chosen SS options between the two groups. This showed a significant difference: *t*(44) = 3.811, *p*<.0001. PG selected 67,73% of SS, whereas CTRL only 54,13% (see Figure 2A), clearly showing a difference in choices as PG displayed a strong bias toward smaller, sooner options.

Then, we computed a mixed ANOVA on reaction times (RTs) as the dependent variable and groups as a between factor. RTs were calculated separately for SS and LL options and for every subject of both groups. This yielded a main effect of reaction time (*F*(1, 43) = 10.118, *p*<.01, as well as an interaction with group (*F*(1, 43) = 4.029, *p*<.05). Independent sample t-tests, corrected for multiple comparisons, confirmed that patients were significantly faster than controls when selecting SS options (*t*(44) = 3.594, *p*<.001), further showing that these smaller, sooner options were selected with greater impulsivity. However, the same difference was not evident when considering LL options (*t*(43) = 1.522, *p* = .135). Within the PG group, the comparison between reaction times when considering the SS and LL options revealed a significant difference (*t*(21) = −4.896, *p*<.0001. This result did not extend to controls (*t*(22) = 0.702, *p* = .49). Note that one subject within the pathological gamblers group was excluded because they selected only SS options. See Figure 2B. This result further confirms that PG are characterized by impatience (faster choices) toward smaller, sooner rewards.

**Figure 3:**
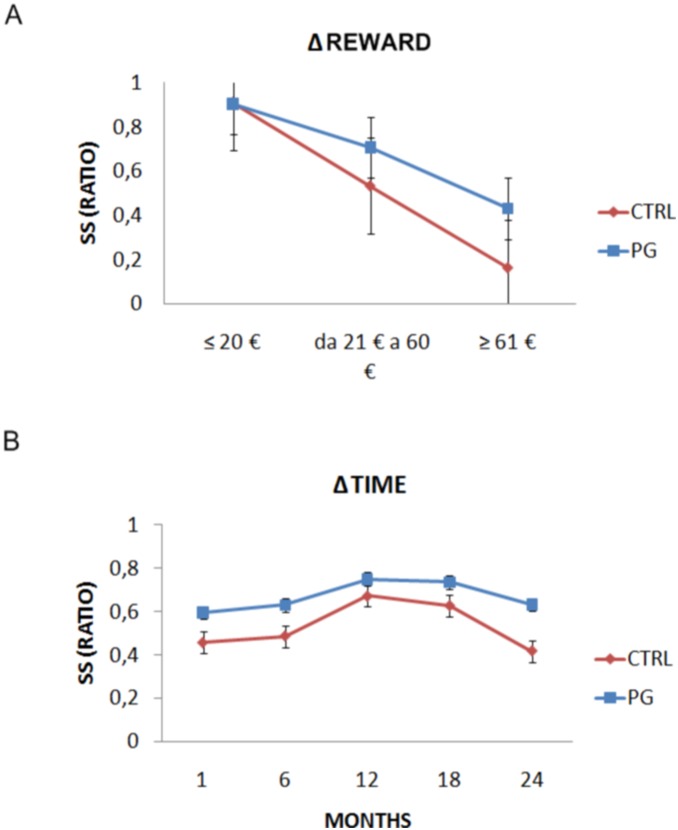
Further performance results. (A) Sooner Small choices are presented separated according to increasing difference in the magnitude of rewards of the two options. Significant differences are displayed between gamblers and controls in the medium and large range. (B) Sooner Small choices are shown for the five months interval., The two groups differed in percentage of SS in all time intervals. Bars indicate stanmdard error of the mean.

Next, we calculated two indices that allowed us to better understand how both the amount of reward and the delay time influenced decisions across both groups. One possibility is that gamblers differ from controls for the way they weight the amount of reward or the amount of waiting time to obtain a reward. Therefore, we calculated the difference between every combination of LL and SS presented during the task (“ΔReward”); then, we separated the percentage of SS choices according to three different ΔReward values, grouped in the following way: LL – SS = 20 € or less (small Δ), from 30 € to 60 € (medium Δ), 70 € or above (large Δ). A mixed Analysis of Variance was fit to the data using number of SS choices as the dependent variable, “ΔReward” (Δ≤20, 30<Δ<60, Δ≥70) as a within-subject factor, and groups (PG vs. CTRL) as a between-subject factor. The factor ΔReward was found to be significant (*F*(2, 88) = 194.85, *p*<.0001), as well as the interaction with groups (*F*(2, 88) = 13.91, *p*<.0001). More specifically, multiple comparisons corrected t-tests showed that patients chose significantly more SS options with medium (*t*(44) = 3.28, *p*<.005) or high (*t*(44) = 5.27, *p*<.0001) ΔReward values. However, they did not differ for small ΔReward quantities (*p*>.008). See Figure 3A. This result confirms that when the amount of reward increases (medium to large) PG are biased toward impatient choices (SS options).

The second calculation involved the factor waiting time, calculated as the difference between waiting time associated with LL and SS options (ΔTime). We split the number of SS choices into the five intervals of delay used in the experimental task: 1, 6, 12, 18 and 24 months of difference between the SS and LL alternatives. A Mixed Analysis of Variance (mixed ANOVA) was fit to the data using the percentage of SS choices as the dependent variable, ΔTime (Δ = 1, Δ = 6, Δ = 12, Δ = 18, Δ = 24) as a within-subject factor, and groups (gamblers vs. controls) as a between-subject factor. Both ΔTime (*F*(4, 176) = 34.73, *p*<.0001), and interaction with groups (*F*(4, 176) = 4.361, *p*<.005) were found significant. Independent sample t-tests corrected for multiple comparisons, showed that the two populations differ when Δ = 1, Δ = 6, Δ = 24 (*p*<.005), but not for Δ = 12, Δ = 18 (*p*>.005). See Figure 3B. In other words, waiting time and amount of reward weight the decisional process of the two populations in different ways. Overall, gamblers prefer SS options, however, they still prefer impatient choices even when the amount of reward increases, whereas controls shift toward a more patient choice (LL options) to increase their gain. The second result is that the two populations differ in terms of choices when the waiting time is very small or very large, with gamblers selecting more SS options as compared with controls.

#### b) Regression analyses

To test the hypothesis that impulsivity, reward amount and delay time all influence participants’ decisions, regression analyses were computed. To begin with, we computed a logistic regression using the three BIS subscales as a measure of general impulsiveness and the two subscales of PG-Y-BOCS (Y-BOCS1 and Y-BOCS2) as measures of impulsivity and gambling compulsiveness the reward amount of the SS option (qSS), the delay time of the SS option (tSS), and Group. The dependent variable was the choice of the SS option (1 = yes, 0 = no). On all the data and findings reported hereafter power analyses were undertaken showing an effect size of 0.6 (medium to large), and a statistical power of 0.9. Results showed that Group was a significant factor (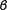 = −2908.508, *p*<.001); the three BIS scales (attention scale, BIS1; motor impulsiveness, BIS2; and non-planning impulsiveness, BIS3) were significant factors in explaining choice (respectively, 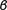 = 8.040, *p*<.001; 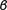 = 7.111, *p*<.001; 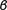 = 5.777, *p*<.001). The same was found for the two scales of PG-Y-BOCS (respectively 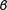 = −247.566, *p*<.001; and 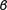 = 309.408, *p*<.001). Both the amount and delay of the of SS were also significant (respectively, 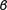 = 0.346, *p*<.001; 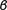 = 0.844, *p*<.05). Notably these factors significantly interacted with the factor Group (Group*BIS1: 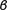 = 246.501, *p*<.001; Group*BIS2: 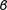 = −49.397, *p*<.001; Group*BIS3: 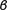 = 106.535 *p*<.001; Group*Y-BOCS1: 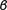 = 292.830, *p*<.001; Group*Y-BOCS2: 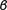 = −46.185, *p*<.001; Group*qSS: 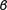 = 0.561, *p*<.001; but not Group*tSS: 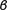 = 0.259, *p* = .655). These results overall confirm a strict relation between impulsivity (as detected by self-report measures) and choices, but also between delay time and amount of reward and choices.

#### c) Delay discounting function

To examine how subjects discounted rewards according to waiting time, we calculated the discount factor. This refers to the decrease in the subjective value of a reward as a function of the delay between the time when an option is chosen and the time when the reward becomes available. The behavior of subjective value over time is well-described through the “hyperbolic model” proposed by Grossbard and Mazur [28], [56]. The discount rate can be described with a family of hyperbolic functions following the above equation:
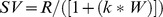



where “SV” summarizes the subjective value of delayed reward. “R” is the value of the reward available after waiting, “W” the waiting time associated with the reward (“R”), and “k” is the discount rate parameter. It provides the effect of reduction on reward (“R”) per 1-unit increase in waiting time (“W”). So, to compute how much someone devalues a quantity over time, the “k” parameter of the hyperbolic function must be derived. This parameter can be calculated by comparing a “SS” options with a “LL”, when the “SS” is chosen, with the following formula:
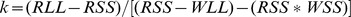
where “RLL” is the reward linked to “LL” option, “RSS” is the reward linked to “SS” option, “WLL” is the waiting time associated to “LL” option and “WSS” is the waiting time of “SS” option. The k value was computed per subject as a mean of the k values calculated for every option that subject selected.

**Figure 4:**
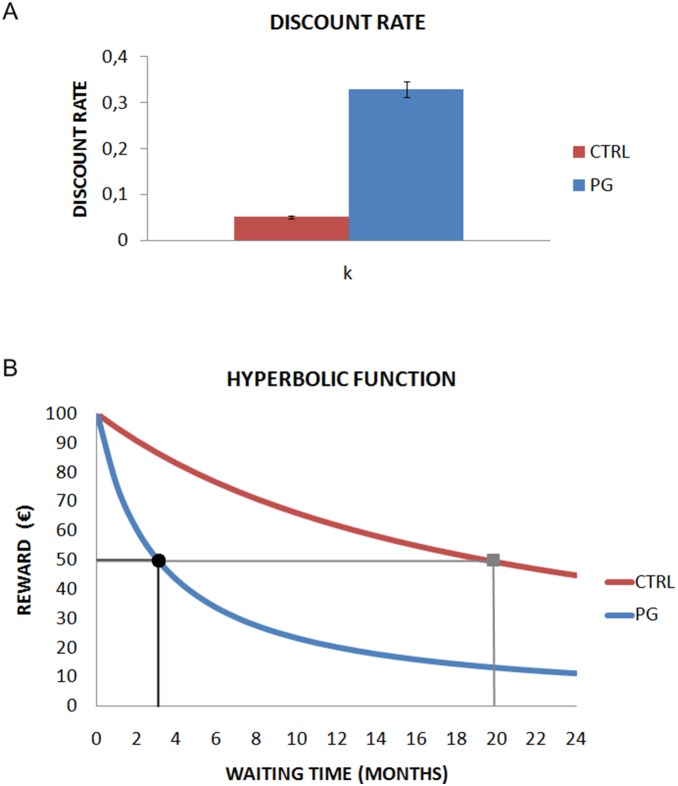
Discount factor and hyperbolic function. (A) Discount factor k is displayed for both groups. Gamblers displayed a larger value about six times larger than controls. (B) Fitted hyperbolic functions are displayed for both groups. Gamblers showed steeper discount function rather than controls. It takes 20 months for controls to discount 100 euros to its half, whereas gamblers show the same result after 3 months.

We found a greater tendency to devalue monetary rewards over time for the PG (k = 0.33) as compared to controls (k = 0.05), *t*(44) = 5.53, *p*<.0001. See Figure 4A. Then, we plotted the hyperbolic function for the two groups according to their k values for a hypothetical reward of 100 € (See Figure 4B). The steepness of the devaluation curve is greater in PG (in blue) than CTRL (in red). We also calculated the area behind the two curves for each participants. T-test revealed a significant difference between controls (area = 732.75) and gamblers (area = 353.86) (*t*(44) = 8.071, *p*<.001).

Additionally, we estimated the “indifference point”, i.e. the point when people choose equally (50%) an SS or an LL option. These points were calculated through comparing the subjective values for the SS and LL alternatives, according to the following equation:
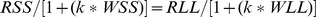



For pathological gamblers, 3.03 months are needed to devalue 100 € to 50 €:
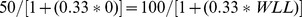


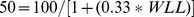


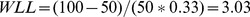
Whereas for controls, 20 months are required to reach the same devaluation:



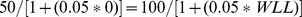


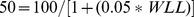


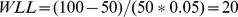



## Discussion

The current study examined how impulsivity may affect decision-making in pathological gamblers as compared to normal controls. Two types of measures were considered: endophenotypical performance measure of reward sensitivity/processing (intertemporal choice task) and phenotypical self-report measures (impulsivity related questionnaires).

At an endophenotypical level, we found that pathological gamblers selected a greater number of smaller sooner choices as compared with age, gender, and education matched non-gamblers. Indeed, when asked to make choices between a smaller sooner option and a larger later one, gamblers were systematically biased toward smaller but more quickly obtainable gains, whereas controls showed no clear preference between the two options (half of the times they selected SS options). Notably, this experiment provides further and more clear evidence that gamblers deviate from controls when making decisions. Previous studies using for example the Iowa Gambling Task failed to find any difference in decision-making when comparing gamblers with healthy controls [57]. Moreover, in our study gamblers were quicker (faster RTs) when selecting the SS option relative to controls, another measure of impatience.

To understand how reward amount and delay time biased participants’ choices, we computed the ratio of SS choices for three intervals of reward magnitude. We found that for both groups when the difference in terms of reward between the options was small (less than 20 euros) they behaved in the same way, that is, selecting with almost 90% the smaller, sooner options. However, when the difference in magnitude increases (medium values), while controls still show no preference between SS and LL (indifference point, 50%), gamblers are biased toward the SS option. Finally, for the higher difference in reward, controls rarely selected the SS (less than 20% of times), while gamblers are still at the indifference point (50%). The same analyses applied to delay revealed that both smaller and larger differences in delays led gamblers to select more SS options as compared with controls. In other words, when the difference in time was small PG selected the sooner available reward, while at the same time when the time to wait was long, they again preferred the smaller, sooner option.

Moreover, both groups were tested for personality traits of impulsivity using the Barratt Impulsivity Scale. At a phenotypical level, the two groups differ in two subscales (motor impulsivity and non-planning impulsiveness), with gamblers scoring much higher values of impulsivity. Another measure of impulsivity and compulsivity more related with gambling behavior used in the present study was the PG-Y-BOCS. Once again, gamblers scored much higher in both subscales. To test for any relation between impulsivity and the quantity of reward and waiting time of the option to be selected, we then computed a regression analyses with factors such as the subscales of BIS, PG-Y-BOCS, the quantity of reward and waiting time of the SS options. This analysis showed a clear and strong relation between impulsivity and choices, but also an effect of reward and time. Importantly, when splitting for the two groups, impulsivity affected both gamblers and controls choices, however, it had a stronger effect on gamblers choices rather than control. In other words, impulsivity affects our intertemporal decisions in normal and abnormal conditions. However, when impulsivity is very high it biases our decisions toward more impatient choices rather when it is lower. We argue that impulsivity may be considered as one of the factors leading subjects to the decision to select the soon available reward.

Bechara [58] suggested a model to explain such “myopia of the future” as a product of an imbalance of two separate but interacting systems that control decision making: an impulsive, intuitive based on the amygdala and other relevant emotional structures (such as striatum) for signaling pain and pleasure of immediate prospects, and a reflective based on the prefrontal cortex for signaling for pain or pleasure for future prospects. In optimal cases, the reflective system has to monitor and eventually inhibit the impulsive system. However, in some cases this does not happen and the impulsive system can override the reflective system. Importantly, the author predicts that the striatum, responding to concrete or abstract monetary rewards, can be one of the forces that may bypass the intervention of the reflective cortical system. Consistent with this hypothesis, the striatum is one of the key regions that seems involved in intertemporal decisions. Neuroimaging studies on normal subjects showed increased striatal activity associated with smaller, sooner choices in the intertemporal task [59], [60]. It should be noted, that fMRI studies on gamblers reported increased activity of the striatum when subjects were presented with gambling cues and monetary gains as compared with controls [61]. Another observation comes from the fact that a double relationship between dopamine (generated by the striatum cells) and impulsivity, and between dopamine and gambling behavior seems to exist. Dopamine is implicated in rewarding and reinforcing behaviors and drug addiction [62]. Indeed, some studies have reported increased cerebrospinal fluid of dopamine and its metabolites in PG [63], [64]. Interestingly, increased dopamine induced by pharmacological administration, led normal subjects to discount more as compared with a placebo condition [60]. Future studies may test for this complex relationship between dopamine, striatum activity and impatient choices as shown by high impulsivity individuals.

Moreover, gamblers discounted in a steeper way as compared with controls, meaning that they devalue monetary rewards over time more rapidly. Notably, we found a similar discount rate for controls as a previous study from Petry [43] and Brevers and colleagues [45] did, even though it is hard to make direct comparisons for obvious differences in the methodology. However, this population has the confound of substance abuse. Unfortunately, Brevers and colleagues [45], did not report k values.

We believe this paper has several implications for the understanding of gambling addiction. Pathological gambling as other addiction disorders are characterized by a lack of self-regulation [20], [65]. Indeed, this condition has been defined as an impulse control disorder by DSM IV-TR [66] and as a behavioral addiction [67], [68] in DSM V [69]. Indeed, previous studies found diminished neurocognitive self-regulatory functions in PG [4], and neurological alterations in reward circuitry [18], [19]. Neurobiological studies showed that diminished dopamine receptor availability (due to addiction behaviors) may cause a chronic reward deficiency in the brain, resulting in a vulnerability to engage in reward seeking behaviors [20]. Indeed, a cardinal feature of gamblers and other addiction behaviors seems to be a tendency to act upon acute impulses, or in other words, they show high traits of impulsivity [70], [71]. However, in the clinical and experimental literature there was not a direct proof of the link between impulsivity and actual decisions inside the population of gamblers. By this experiment we were able to show not only that gamblers do suffer from high impulsivity, but more importantly, that such impulsivity affects how they decide upon an immediate available options (leading to impatient choices). This behavior can explain why these individuals engage in available bets without the possibility to inhibit nor postpone them. Indeed, recent experimental observations led scientists to consider disinhibition as a predominant executive deficit present in PG. For example, one study from Potenza et al, [18] showed that in a Stroop task, gamblers compared to controls, were showing less activation in inhibition areas such as the left middle and superior frontal gyri. A recent review examined several performance measures of reward sensitivity/processing of inhibitory processes such as filtering of irrelevant information and inhibiting prepotent responses that are impaired in gamblers [61]. These neurocognitive impairments seem coherent with a lack of impulse control or high impulsivity when presented with real or hypothetical rewards.

In more cognitive terms, a distinction between two kinds of thinking, often termed reflective and intuitive, has been proposed by some researchers to account for such phenomena [72], [27]. Intuitive thinking is typically described as quick, emotion-based and with no conscious effort required; reflective thinking is slow, norm-based and conscious. Authors have suggested that these two ways of thinking can be seen as different types of processes (Type 1 and 2). Evans [72], conceptualized the conflict between these two styles of thinking as a “cognitive control problem”, referring to the fundamental question of the mechanism by which control over the answer is ultimately allocated, and suggested how this conflict could be resolved. He points out that when confronted with a decision, Type 1 processing automatically produces default intuitive responses, unless the slower and more reflective Type 2 processing intervenes (see also [27]). This view is coherent with the neurobiological view of a diminished neurocognitive self-regulatory functions in PG [4], [73].

Beside the novelty of the study, some limitations must be acknowledged. Even though the control group was matched to PG group for relevant dimensions, we must acknowledge the fact that other demographical and social status factors were not taken into account. Future studies may want to control also for these factors. Another limitation relies in the fact that the intertemporal choice paradigm we used was quite different from the ones used by other authors in the past, thus limiting a direct comparison between results. At a more conceptual level, the definition of impulsivity seems strongly correlated with the concept of lack of inhibition. Indeed, one definition of impulsivity is the lack of inhibition. However in the present study the link between lack of inhibition and decisions has not been tested. Future studies may want to address this topic by using response inhibition tasks such as the stop-signal paradigm [74] to test the hypothesis that gamblers have deficits in this domain and that these deficits are related with impulsive decisions. Notably, response inhibition is connected with the fronto-basal-ganglia circuit that has been shown to have implications for addiction problems. Last but not least, we acknowledged the fact that PG were tested inside the clinic in which they were admitted for treatment and not in their natural gambling context (see [49] for the effect of context on gambling behavior). This may have biased the results. In conclusion we believe that this study sheds some light on the link between impulsivity and impatient decisions the population of pathological gamblers. Moreover, we believe this study can add understanding on the etiology of behavioral addictions and impulse control disorders, and may offer some indications for clinical interventions on this relevant and nowadays widespread pathology. One consequence of these results is that if clinicians want impulsive choices to be reduced, impulsivity must be contained and regulated. Moreover, knowing that gamblers suffer from strong biases in their decision-making and in the temporal perception of rewards, can greatly help clinicians to incorporate methods and techniques to balance decision based biases in their clinical work.
